# Potential clinical implications of *BRAF* mutations in histiocytic proliferations

**DOI:** 10.18632/oncotarget.2061

**Published:** 2014-06-06

**Authors:** Anna-Maria Bubolz, Stephanie E. Weissinger, Albrecht Stenzinger, Annette Arndt, Konrad Steinestel, Silke Brüderlein, Holger Cario, Anneli Lubatschofski, Claudia Welke, Ioannis Anagnostopoulos, Thomas F. E. Barth, Ambros J. Beer, Peter Möller, Martin Gottstein, Andreas Viardot, Jochen K. Lennerz

**Affiliations:** ^1^ Institute of Pathology, University Ulm, Ulm, Germany; ^2^ Institute of Pathology, University Hospital Heidelberg, Heidelberg, Germany; ^3^ Institute of Pathology and Molecular Pathology, Bundeswehrkrankenhaus Ulm, Ulm, Germany; ^4^ Bundeswehr Institute of Radiobiology, Munich, Germany; ^5^ Department of Pediatric Oncology, Children's Hospital, University Ulm, Ulm, Germany; ^6^ Comprehensive Cancer Center Ulm, Ulm Germany; ^7^ Institute of Pathology, Charité University Hospital, Berlin, Germany; ^8^ Department of Nuclear Medicine, University Ulm, Ulm, Germany; ^9^ Department of Internal Medicine III, University Ulm, Ulm, Germany

**Keywords:** Langerhans, Biomarker, Erdheim-Chester, V600E

## Abstract

For a growing number of tumors the *BRAF* V600E mutation carries therapeutic relevance. In histiocytic proliferations the distribution of *BRAF* mutations and their relevance has not been clarified. Here we present a retrospective genotyping study and a prospective observational study of a patient treated with a BRAF inhibitor.

Genotyping of 69 histiocytic lesions revealed that 23/48 Langerhans cell lesions were *BRAF*-V600E-mutant whereas all non-Langerhans cell lesions (including dendritic cell sarcoma, juvenile xanthogranuloma, Rosai-Dorfman disease, and granular cell tumor) were wild-type. A metareview of 29 publications showed an overall mutation frequency of 48.5%; and with N=653 samples, this frequency is well defined. The *BRAF* mutation status cannot be predicted based on clinical parameters and outcome analysis showed no difference. Genotyping identified a 45 year-old woman with an aggressive and treatment-refractory, ultrastructurally confirmed systemic *BRAF*-mutant LCH. Prior treatments included glucocorticoid/vinblastine and cladribine-monotherapy. Treatment with vemurafenib over 3 months resulted in a dramatic metabolic response by FDG-PET and stable radiographic disease; the patient experienced progression after 6 months.

In conclusion, *BRAF* mutations in histiocytic proliferations are restricted to lesions of the Langerhans-cell type. While for most LCH-patients efficient therapies are available, patients with *BRAF* mutations may benefit from the BRAF inhibitor vemurafenib.

## INTRODUCTION

Histiocytic tumors are derived from the mononuclear-phagocytic and histiocyte system[1-3]. The clinical manifestations of the currently recognized disease entities are highly variable and range from benign localized lesions to highly aggressive systemic diseases[4]. A distinction between lesions that share surface markers of Langerhans cells (LCH-like lesions) from those that carry other surface markers of the histiocytic or dendritic cell lineage has been adopted[1]. Briefly, the latter group is composed of various entities that can be summarized as non-LCH-type tumors and is reviewed elsewhere[1, 5]. Due to the lack of robust evidence from randomized controlled clinical trials, therapeutic strategies currently rely on precise histotyping as well as disease classification by involved system[6]. For example, therapeutic regimens for patients with systemic involvement include radiation and/or chemotherapy and, more recently, early evidence for molecular targeted approaches[7-9].

**Figure 1:**
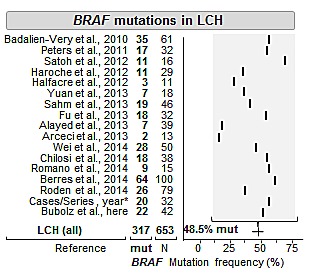
Metareview of Reported Mutation Frequencies and our *BRAF* Genotyping Results in Langerhans cell histiocytosis *A total of 13 case reports/series are summarized (see methods). *Abbreviations:* LCH, Langerhans cell histiocytosis; mut., mutated; N, number of tested samples. In the summary line, the average of all studies is provided along with the 95% confidence interval assuming a binomial distribution for all included cases (line).

In 2010 *BRAF* mutations have been described in a significant number of LCH patients[10]. The biological role of *BRAF* mutations at that time was seen as an oncogenic driver leading to constitutive activation of the RAS/RAF pathway[11-13]. Notably, recent trials (e.g. in melanoma) indicate that pharmacological interference using second-generation BRAF inhibitors produces sustained tumor regressions[14, 15]. Although the original study from 2010 described *BRAF* V600E mutations in 57% of LCH patients, it is only now that this finding received the appropriate level of attention and several other groups confirmed it (Figure 1). The specific diagnostic value of *BRAF* mutations in LCH has still not been thoroughly determined. As for now there is no statistical evidence that the *BRAF* mutation correlates with affected site or outcome[10, 16-18]. A dramatic response to targeted BRAF inhibition in three patients with refractory Erdheim-Chester has been reported[18], implying therapeutic relevance although data in classic LCH and confirmation by an independent group are currently lacking.

We performed a study consisting of three parts: a) a literature review of *BRAF* mutations in LCH, b) a retrospective genotyping study in a series of histiocytic tumors. The identified patients were screened for being refractory to established treatment regimens and we additionally report c) the prospectively observed transient response to the targeted BRAF inhibitor vemurafenib in one patient. Our data indicate that *BRAF* mutation testing of tumor tissue should be carried out in LCH patients refractory to conventional treatment to identify those patients that may benefit from the salvage therapeutic option of BRAF inhibition.

## RESULTS

Mutation analysis was performed in 69 patient samples and genotyping results by histological subtype are summarized in Table 1. We found no *BRAF* mutations in the 21 tested non-Langerhans cell lesions. Briefly, by histological subtype, our findings in juvenile xanthogranuloma and Rosai-Dorfman disease are in accord with a prior report[19]. We also genotyped three follicular dendritic cell tumors that were wild-type, which has not been previously examined; however, a *BRAF* V600E mutant interdigitating dendritic cell sarcoma has been recently reported[20]. The additionally tested granular cell tumors were all wild-type, which has to our knowledge not been previously examined.

**Table 1:** Results of *BRAF* Genotyping by Histological Subtype

Histological subtype	Total	*BRAF*^V600E^	
	N=69	No.	%
Tumors derived from Langerhans cells	48	23	48
Langerhans cell histiocytosis	42	22	52
Solitary/unifocal	25	12	48
Multi-system	13	7	54
Disseminated/visceral	4	3	75
Langerhans cell sarcoma	6	1	17
Tested Non-Langerhans cell entities	21	0	0
Dendritic cell sarcoma	3	0	0
Juvenile Xanthogranuloma	3	0	0
Rosai-Dorfman Disease	4	0	0
Granular Cell Tumor	11	0	0

Abbreviations: N, number of cases, No., number of mutated cases.

**Figure 2:**
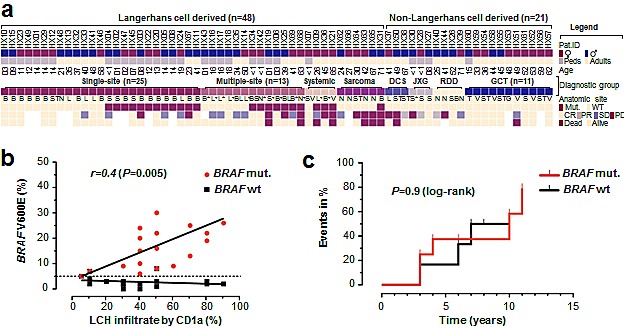
*BRAF* Mutation Analysis at the Case Level and Correlation with Tumor Cell Density and Event Rate in Non-Responders **a**. Key clinicopathological features of individual patients along with genotyping result and coded outcome data. Samples displayed as columns and arranged by disease entity, *BRAF* mutation status and age. **b**. Correlation of histiocytic infiltrate (as determined by CD1a staining) with peak-height quantification from pyrosequencing in 19 *BRAF* V600E mutant and 19 wild-type LCH cases. **c**. Stacked cumulative event rate (stable disease, recurrence, progression) in patients with multi-site or multi-system disease according to the *BRAF* mutation status. *Abbreviations*: ^*^, multiple sites; ^+^, central nervous system/additional organ involved; B, bone; BS, bone and skin; CR, complete response, DCS, dendritic cell sarcoma; JXG, juvenile xanthogranuloma; G, genital; GCT, granular cell tumor; L, lung; LCH, Langerhans cell histiocytosis; N, lymph node; PD, progressive disease; PR, partial response; RDD, Rosai-Dorfman, disease; S, skin; SD, stable disease; ST, soft-tissue; T, tongue; V, visceral.

Genotyping in the 48 patient-samples with Langerhans cell-derived lesions identified a total of 23 *BRAF* V600E mutations (=47.9%). Comparison of detection rates with our literature meta-review findings demonstrated that our detection rate is in line with prior reports and our cohort representative. With over 600 tested cases, enough cases have been reported and the mutation frequency of 48.5% (95% confidence interval: 44.7-52.5%) can now be considered well-defined (Figure 1). Although *BRAF* variant mutations in LCH have been reported (e.g., V600D)[21], we did not observe such variant mutations in our series. The fractions of mutant LCH-cases were relatively constant between different histological subtypes; however, we noted two exceptions. First, of the 6 tested Langerhans sarcomas we found only one *BRAF* V600E mutation in a 71 year-old male who died 3 months after diagnosis (Figure 2a). Second, we found *BRAF* mutations in all 4 tested solitary cutaneous LCHs. Despite the small number of tested samples, these findings raise the question whether the *BRAF* mutation status can distinguish between solitary and multiple-site or systemic disease.

To explore the diagnostic value of *BRAF* genotyping in LCH, we were interested whether quantification of mutant peak heights (by pyrosequencing) can function as a surrogate marker for the estimation of histiocytic infiltrates. We found significant correlation between mutant peak heights and CD1a staining (Figure 2b); thus indicating a robust relationship between total mutated allele content and CD1a-positive infiltrate. Additional diagnostic value may be related to the clinicopathological phenotype; however, we failed to detect significant associations with age, sex, ‘stage’ (i.e. system involvement) or affected organ/site (Table 2). To corroborate these findings we also re-tested the diagnostic distinction for skin, bone, and lung lesions based on *BRAF* genotype and found no significant associations (Table 2). For example when confronted with a LCH in a lung biopsy, we tested whether the *BRAF* status can distinguish between local vs. systemic disease. Comparison of our 7 lung samples from patients with systemic involvement vs. 4 ‘lung-only’ cases showed that there was a slightly higher mutation frequency in systemic (2 of 7 = 28.6%) vs. localized cases (1 of 4 = 25%); however, these differences did not reach statistical significance (*P*=1.00; Fisher's). Collectively, these findings suggest a limited value of *BRAF* testing to separate single-site from multi-site or multi-system LCH.

**Table 2:** Demographic and Clinical Characteristics of Genotype-Specific Subsets of Patients with Langerhans cell Histiocytosis

		*BRAF* mutated (n=22)		*BRAF* Wild-type (n=20)	P
Characteristic	No.	%	No.	%	
Age, years					
Median	13		15		0.68
Range	0.6-65		0.6-57		
Sex					
Male	10	45	12	60	0.37
Female	12	55	8	40	
Stage					
Single-System	12	55	13	65	0.54
Multi-System	10	45	7	35	
Affected Site					
Bone (n=23)					
Solitary (n=17)	9	53	8	47	0.66
Multi (n=6)	4	67	2	33	
Lung (n=11)					
Solitary (n=4)	1	25	3	75	1.0
Multi (n=7)	2	29	5	71	
Skin (n=6)					
Solitary (n=4)	4	100	0	0	0.33
Multi (n=2)	1	50	1	50	

Abbreviations: n=total number of cases; No., number of cases per characteristic; P-values derived from contingency testing (t-test for age; Fisher's exact test for dichotomous factors); solitary, represents single-site involvement; multi, represents multi-system disease; Note: in the „affected site“ part of the table, percentages represent the fraction of mutated or wild-type cases in each category (line-wise comparisons).

Given the relatively high rate of *BRAF* mutations in multi-system LCH – for example with bone involvement (n=4/6=66.6%), the mutation status may hold prognostic information. Therefore, we compared overall and progression free survival between *BRAF* wild-type and mutant patients; however, found no significant difference (*P*=0.68 PFS; *P*=0.37 OS; log-rank). In addition, we examined whether the *BRAF* mutation status can distinguish between responders and non-responders. In the 18 mutant cases with outcome information, we found 12 non-responders (66.6%) whereas there were 5 non-responders among the 15 wild-type cases with outcome information (33.3%); however, this difference did not reach significance (*P*=0.084; Fisher's exact). Thus, the *BRAF* mutation status does not allow stratification of either responders or non-responders; at least not in our cohort. In addition, we examined whether the *BRAF* mutation status is associated with a different time course of events (e.g. progress, recurrence) in the non-responder subgroup. Event plots show no difference in the time course when non-responders were separated according to genotype (Figure 2c). Together, our outcome analysis indicates that the *BRAF* mutation status holds –at this point– no prognostic information.

**Figure 3:**
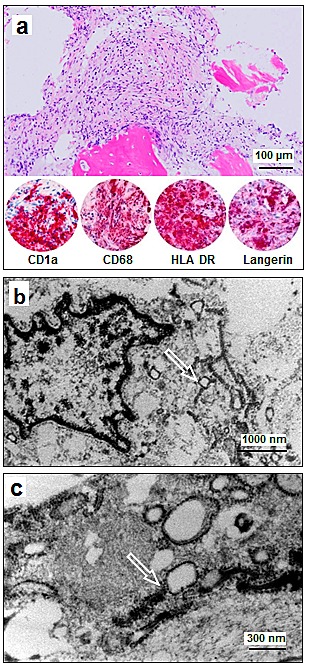
Histology, Immunophenotype and Ultrastructural Findings in Patient HX 36 **a**. Bone marrow biopsy sample shows a dense histiocytic infiltrate with reactive resorption of trabecular bone and replacement of the bone marrow. Morphology and immunophenotype (selected images of the immunophenotype are provided) are diagnostic of LCH (H&E and alkaline phosphatase immunohistochemistry). **b**: Ultrastructural examination of the histiocytic infiltrate shows lobulated nuclei with open chromatin, lack of prominent nucleoli, and tennis-racket shaped cytoplasmatic Birbeck granules (arrow). **c**: Ultra high magnification of Birbeck granules shows the rod shaped electron dense configuration and reveal vacuolated blebs, diagnostic of LCH (arrow).

**Figure 4:**
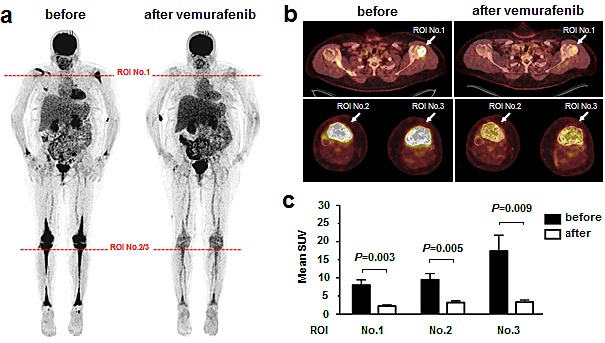
Response of LCH to the *BRAF* Inhibitor Vemurafenib Shown are images pretreatment and after 6 weeks. a: Pretreatment FDG-PET maximum intensity projection (MIP) images before and after 6 weeks of treatment with vemurafenib 240mg/day for 1 week and vemurafenib 480mg/day for 5 weeks. Horizontal lines at full body scan indicate planes for humoral head and tibia plateau cross sections. b. PET-CT fusion images of humoral head and tibia plateau. Arrows indicate region of interest (ROI). c. Quantitative comparison of mean standardized uptake values (+/− standard deviation); *P*-values from unpaired t-test.

Due to the compelling rate of *BRAF* mutations in LCH, we started *BRAF* testing in therapy refractory LCH patients as a prospective observational study component. At time of review, 10 of the 17 non-responders were lost to follow up, 4 had died and only 3 non-responders were alive, and required additional treatment. Two of the three patients were children (one *BRAF* mutant; who received second-line chemotherapy) and the third patient was a 45-year-old woman with multifocal and multisystemic *BRAF* V600E mutant Langerhans cell histiocytosis with multiple bone lesions (Patient HX36). Diagnosis in patient HX36 was established via histology and determination of the typical immunophenotype. The patient also had orbital and meningeal lesions and infiltration of the pituitary gland and hypothalamus without diabetes insipidus. The patient also had a severe insulin-dependent diabetes mellitus with normal cortisol and ACTH-levels. Given the high similarity of affected sites in our patient to the previously reported patients with Erdheim Chester disease[18] we performed electron microscopic analysis (Figure 3b, 3c) and found Birbeck granules in ultrastructural examination thereby corroborating the diagnosis of Langerhans cell histiocytosis. Consequently, the woman was treated with glucocorticoids (prednisolone up to 1mg/kg) and vinblastine (for one month; repeated 4 months later), which was not tolerated due to aggravation of the diabetes mellitus, and which resulted in a progressive disease with new intracranial and meningeal manifestations. Therapy was switched to cladribine monotherapy (2,1mg/m² day 1-5; 2 cycles; for three months), which again resulted in progressive disease with new intracranial lesions. Therefore, we decided to start off-label use of vemurafenib with escalating doses over time (240mg once daily for one week, 240mg twice daily for 5 weeks and 240mg four times a day for another 6 weeks). Overall, treatment was well tolerated. Skin examinations were performed during and after the treatment. The patient had a reduced need for analgesics for the bone lesions, and a slightly lower need for insulin. After 6 weeks (at the lower dosage of 2x 240mg daily), the patient displayed an almost complete metabolic remission in the FDG-PET, without significant reduction of the size of measurable lesions (e.g. hypophyseal-hypothalamic infiltration). After 3 months, the patient still had stable disease according to RECIST criteria with a continued, dramatic metabolic response (Figure 4). Due to a lack of published data, the off-label use had to be discontinued and 6 months later, the patient had progressive disease.

## DISCUSSION

In our analysis, *BRAF* V600E mutations in histiocytic proliferations are restricted to tumors of the Langerhans cell lineage. Of the few refractory patients we identified a patient with a *BRAF* mutant LCH who had a transient clinical response to the targeted BRAF inhibitor vemurafenib.

The frequency of *BRAF* mutations in LCH approximates 50% (Figure 1) and raises the question how these genetically distinct tumors differ from LCH wild-type tumors. To this end we performed phenotype comparisons (Table 2); however, with two exceptions we failed to identify distinctive features. These exceptions were: the presence of *BRAF* mutations in all solitary skin LCHs and a somewhat lower rate of *BRAF* mutations in Langerhans cell sarcomas (17%). Due to the small number of tested samples in these categories, we abstain from speculations; however, would like to point out two aspects of these orphan-diseases. First, our *BRAF* mutated Langerhans cell sarcoma patient had died; therefore, given the rarity and aggressiveness of this disease the potential of targeting *BRAF* mutations should not be ignored. Second, the distinction of localized vs. multi-system disease remains a diagnostic challenge[22]. In particular, in the setting of a histiocytic lung lesion we assessed the value of the *BRAF* mutation status as a diagnostic biomarker but did not succeed in finding a statistical difference between mutated and wild-type cases. Similarly, the mutation frequency with respect to the involved system did not show any differences. In conjunction with the available literature, our findings indicate that neoplastic lesions related to LCH (e.g., Erdheim-Chester disease)[19, 23] also harbor *BRAF* mutations. Nonetheless, the specific diagnostic value of *BRAF* in LCH seems not as straightforward as for example in hairy cell leukemia[24]; although very recent evidence suggests distinct subtypes of Langerhans cell histiocytosis depending on the presence or absence of circulating dendritic cells[16], which could be used for therapeutic monitoring. However, there is currently no convincing evidence that the *BRAF* status by itself has value as a prognostic biomarker; at least not in our series of non-responders (Figure 2c). Given recent data describing somatic activating *ARAF* mutations in *BRAF* wild-type LCH[25], it is expected that the advents of novel sequencing technologies will lead to a more comprehensive delineation of the mutational landscape in LCH.

Finally, the therapeutic relevance of *BRAF* mutations in LCH has not been clarified and may differ between tumor types. Here we add the transient response of one LCH patient who demonstrated at least partial metabolic response. Besides adding this case as proof of principle in LCH, there are three issues worth noting. First, despite the classical histological, immunophenotypic and ultrastructural findings (that were all diagnostic of LCH), the clinical presentation and distribution of the disease is highly similar –if not identical– to a patient with Erdheim-Chester disease reported by Haroche et al.,[18]. Second, while the formal criteria for a radiographic partial response (by RECIST) were not met, achieving a transient response in such an aggressive version of LCH is striking and we find the metabolic response at least noteworthy (Figure 4). The similarities of our data and those from xenograft models are impressive[26] and indicate a metabolic function of mutant-*BRAF* in histiocytic neoplasia[26-31]. We interpret the stable radiographic disease as an apparent lack of ‘oncogenic shock’[32, 33]. Third, from an academic perspective, a re-biopsy would have been interesting; however, clinically this was not indicated and the location of lesions was considered “high-risk” (e.g. intracranial). Collectively, the efficacy of vemurafenib beyond melanoma[34], for example in Erdheim-Chester Disease[18] and our data argue for ongoing exploration of the therapeutic value of *BRAF* mutations in LCH.

In summary, *BRAF* mutations in histiocytic proliferations are restricted to those of the Langerhans cell-type and our metareview establishes the *BRAF* mutation frequency of 48.5%. In addition, for the vast majority of LCH-patients efficient therapeutic options are available; however, for treatment refractory patients with lesions harboring the *BRAF* V600E mutation, targeted BRAF inhibition may represent a therapeutic option.

## MATERIAL AND METHODS

### Design and Ethical Approval

The retrospective part of the study was conducted as an anonymized case- and specimen review via the Comprehensive Cancer Center Ulm (CCCU). The prospective interventional study in one patient included informed consent for experimental off-label drug treatment. All experiments were performed in accordance with the ethical standards of the local ethics committee and with the Declaration of Helsinki.

### Study Cohort, Tissue Sections, Diagnostic Criteria

We searched the pathology- and the hospital information systems using diagnostic terms and ICD-O codes. After removal of duplicates, samples with tissue available for molecular genetic analysis were reviewed. Atleast two board-certified pathologists confirmed each primary diagnosis by review of original sections and immunohistochemical stains. Diagnostic criteria followed the 2008 WHO guidelines.

### Microdissection and DNA extraction

Regions for microdissection were identified by immunohistochemistry and H&E stains. The tumor regions were either sectioned (at 2-5μm thickness) and microdissected, or cored using a 2 mm dermal punch-biopsy needle. After deparaffinization and DNA extraction, concentration was determined using an Ultrospec 2100pro (Amersham Biosciences; Upsala; Sweden).

### Molecular Genetic Analysis

PCR-reactions using the primers: F-5'-TGC-TTG-CTC-TGA-TAG-GAA-AAT-G-3' and R-5'-AGC-ATC-TCA-GGG-CCA-AAA-AT-3', were followed by pyrosequencing [R-5-GAC-CCA-CTC-CAT-CGA-G-3; PyroMark Q24 (Qiagen, Hilden, Germany)] according to established protocols [35]. Alternatively, mutation analyses was performed using the *BRAF* 600/601 StripAssay (Vienna Labs, Vienna, Austria) according to the manufacturer's instructions. Mutations were validated using conventional Sanger sequencing.

### Electron Microscopy

Electron Microscopy was performed according to established protocols [36]. Briefly, FFPE tissue was microdissected, deparaffinized and post-fixed overnight at 4°C in modified Karnovsky's fixative (3% glutaraldehyde in 1% paraformaldehyde in sodium cacodylate buffer at pH 7.4). After rinsing in sodium cacodylate buffer, samples were postfixed in phosphate cacodylate-buffered 21% OsO_4_ for 1h, dehydrated in graded ethanol with a final dehydration in propylene oxide, and embedded in epon (Electron Microscopy Sciences, Hatfield, PA). One-micron-thick plastic sections were examined by light microscopy after being stained with toluidine blue. Ultrathin sections (90 nm thick) were cut and mounted onto slot grids. Sections were poststained with uranyl acetate and lead citrate and viewed with an EM10 (Zeiss, Jena, Germany). Digital images were acquired using the TVIPS 1k system (TVIPS, Gauting, Germany).

### Literature review

Two electronic databases (Medline, Scopus) were searched (Jan 1976 – Feb 2014) independently by three authors (AMB/SEW/JKL) using the search terms histiocy*, langerhans*, erdheim*, bone or *lung* in combination with *BRAF* or *V600E*. In addition, we performed review of reference lists in all selected articles to identify additional studies that contained *BRAF* mutational analysis. For each study [10, 16, 18, 19, 37-48], we noted histological subtype of the lesion, *BRAF* mutation status, and the total number of mutated and tested cases. We present the overall *BRAF* mutation frequency along with the 95% confidence interval assuming a binomial distribution for all included cases. For simplicity of display we summarized case reports and small series that contained less or equal to ten patients in one category (Figure 1) [19-21, 49-58].

### Outcome and Statistical Analysis

Outcome analysis consisted of four elements: a) overall- and b) progression free survival defined as the time interval between diagnosis and death or recurrence/progression; c) we determined the fraction of responding and non-responding patients and d) determined the time course of the events in the non-responding subgroup. We defined non-responders as those patients with stable disease, progressive disease or relapse (‘events’) whereas patients with partial or complete response were considered responders. For a time to event analysis, we defined the timeframe from initial diagnosis to first event, which we plotted as stacked event curves that we compared by using a log-rank test. For statistical comparisons of mutation frequencies we used the Fisher's exact- or Chi-square test, age comparisons employed the student's t-test, and we considered *P*<0.05 as statistically significant.
